# Serum Cocaine- and Amphetamine-Regulated Transcript (CART) Levels in Graves’ Disease: Associations with Metabolic Status, Autoimmunity, and Thyroid Ultrasound Heterogeneity

**DOI:** 10.3390/ijms27052428

**Published:** 2026-03-06

**Authors:** Betül Çiğdem Yortanlı, Ümmügülsüm Can, İslam Köse, Semiha Durmaz, Mehmet Yortanlı, Oğuzhan Aksu

**Affiliations:** 1 Department of Internal Medicine, Konya City Hospital, University of Health Sciences, Konya 42020, Turkey; semiha.simsek2@saglik.gov.tr; 2 Department of Biochemistry, Konya City Hospital, University of Health Sciences, Konya 42020, Turkey; ummugulsum.can@saglik.gov.tr (Ü.C.); islam.kose@saglik.gov.tr (İ.K.); 3 Department of Emergency Medicine, Konya Numune Hospital, Konya 42060, Turkey; mehmet.yortanli@saglik.gov.tr; 4 Division of Endocrinology and Metabolism, Department of Internal Medicine, Konya City Hospital, University of Health Sciences, Konya 42020, Turkey; oguzhan.aksu@saglik.gov.tr

**Keywords:** Graves’ disease, autoimmune diseases, thyroid autoantibodies, thyroid diseases, hyperthyroidism, endocrinology, CART, biomarkers

## Abstract

Graves’ disease (GD) is an autoimmune disorder characterized by hyperthyroidism and a hypermetabolic state involving complex endocrine, metabolic, and immune interactions. Cocaine- and amphetamine-regulated transcript (CART) is a neuropeptide involved in energy balance, neuroendocrine signaling, and neuroimmune modulation; however, its circulating levels and clinical relevance in GD remain unclear. In this single-center prospective study, serum CART levels were evaluated in 44 patients with GD and 44 age- and sex-matched healthy controls. Associations with thyroid function, autoimmune markers, metabolic parameters, and thyroid ultrasound heterogeneity were analyzed. Serum CART concentrations were measured using an enzyme-linked immunosorbent assay, and clinical, biochemical, and ultrasonographic data were recorded. Serum CART levels did not differ significantly between GD patients and healthy controls. However, within the GD group, CART levels varied significantly according to thyroid ultrasound heterogeneity, with lower levels observed in patients with severe parenchymal heterogeneity. Serum CART levels showed positive correlations with body mass index and insulin resistance indices, while inverse correlations were observed with thyrotropin receptor antibody and anti-thyroid peroxidase antibody levels. No significant associations were identified between serum CART levels and circulating thyroid hormone concentrations. These findings suggest that serum CART may reflect metabolic and autoimmune heterogeneity rather than hypothalamic–pituitary–thyroid axis activity in GD, supporting its role as a context-sensitive, hypothesis-generating biomarker.

## 1. Introduction

Graves’ disease (GD) is the most common cause of hyperthyroidism of autoimmune origin and is caused by stimulatory autoantibodies directed against the thyrotropin receptor (TRAb). It is characterized by elevated thyroid hormone levels, suppressed thyrotropin (TSH), and a pronounced hypermetabolic state. Excess thyroid hormones lead to numerous systemic effects, including increased energy expenditure, weight loss, enhanced activation of the sympathetic nervous system, and alterations in glucose metabolism. Therefore, GD is considered not only a thyroid disorder but also a condition that affects central and peripheral metabolic regulatory mechanisms [1,2].

Thyroid hormone secretion is tightly regulated through the hypothalamic–pituitary–thyroid (HPT) axis. Thyrotropin-releasing hormone (TRH), the central regulator of this axis, is synthesized in the paraventricular nucleus (PVN) of the hypothalamus and stimulates TSH secretion from the anterior pituitary. In recent years, it has been shown that the activity of TRH neurons is modulated not only by the feedback effects of thyroid hormones but also by neuropeptides associated with physiological states such as energy balance, fasting, cold exposure, and stress [1,2,3].

In this context, cocaine- and amphetamine-regulated transcript (CART) has attracted attention as an important neuropeptide that is widely expressed in the central nervous system and exerts appetite-suppressing and energy expenditure-increasing effects [4,5]. CART has been shown to densely innervate hypophysiotropic TRH neurons in the hypothalamus, particularly within the PVN, and to be co-expressed with TRH in a substantial proportion of these neurons [6,7,8]. Experimental studies have demonstrated that CART can stimulate TRH gene expression and may therefore play a role in the adaptation of the HPT axis to physiological conditions [5,6].

In hypermetabolic states such as hyperthyroidism, there is a marked overlap between the physiological effects of CART on increasing energy expenditure and suppressing appetite and the associated clinical manifestations. However, although the effects of CART have largely been characterized through the central nervous system, the relationship between circulating (serum) CART levels and GD in humans, as well as their associations with autoimmunity, thyroid hormones, and metabolic parameters, has not yet been sufficiently elucidated [4,5].

In this study, an exploratory approach was adopted to evaluate serum CART levels in GD, to compare them with those of healthy individuals, and to examine the associations between CART levels and thyroid hormones, autoantibodies, and metabolic markers. This approach is expected to provide preliminary findings regarding the potential role of CART in GD and to offer hypothesis-generating data on neuroendocrine and metabolic adaptation mechanisms in hyperthyroidism.

## 2. Results

**Figure 1:**
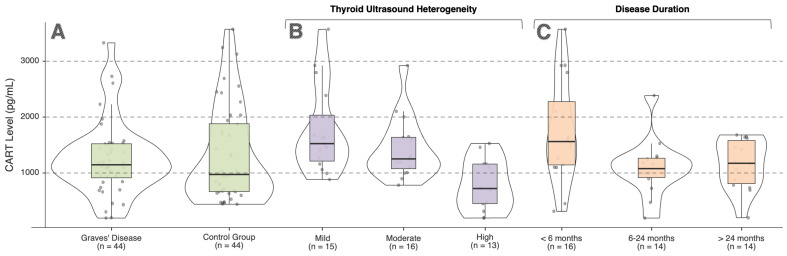
Distribution of serum cocaine- and amphetamine-regulated transcript (CART) levels in patients with Graves’ disease and healthy controls. (**A**) Comparison between Graves’ disease and healthy controls. (**B**) Distribution of serum CART levels according to thyroid ultrasound heterogeneity. (**C**) Distribution of serum CART levels according to disease duration. Boxes represent median and interquartile range, and whiskers indicate minimum and maximum values. Individual dots represent single participants.

A total of 88 participants were included in the study, comprising 44 patients with GD and 44 healthy controls. There was no significant difference between the groups in terms of sex distribution (*p* = 0.826). No statistically significant differences were observed between the groups with respect to age, height, or body weight. Body mass index (BMI) values were higher in the GD group compared with the control group (*p* = 0.044). There were no significant differences between the groups in fasting plasma glucose, glycated hemoglobin (HbA1c), insulin levels, or insulin resistance (HOMA-IR). Evaluation of thyroid function tests showed higher free triiodothyronine (fT3) and free thyroxine (fT4) levels and lower TSH levels in the GD group (all *p* < 0.001). Serum CART levels were similar between the GD and control groups (*p* = 0.553) (Table 1, Figure 1A).

Among GD patients classified according to thyroid ultrasonography, a significant difference in serum CART levels was observed among the ultrasound heterogeneity groups (*p* < 0.001). CART levels were higher in the mild heterogeneity group and lower in the high heterogeneity group. Post hoc analyses showed that CART levels in the high heterogeneity group were significantly lower than those in both the mild and moderate heterogeneity groups (*p* = 0.002 and *p* = 0.006, respectively). In the same grouping, a significant difference was also detected in TRAb levels (*p* = 0.003). No significant differences were observed among the ultrasound heterogeneity groups with respect to other clinical and biochemical parameters (Table 2, Figure 1B).

In comparisons based on disease duration, no statistically significant difference in serum CART levels was observed among the three groups (*p* = 0.054). TRAb levels differed according to disease duration (*p* = 0.044); post hoc analyses indicated that this difference was between the 6–24-month group and the >24-month group (*p* = 0.047). No significant differences were observed among the groups for the other parameters (Table 3, Figure 1C).

Among tertile groups defined according to serum CART levels in the entire study population, significant differences were observed in height (*p* = 0.003) and BMI (*p* = 0.010). Post hoc analyses indicated that the significant differences in both height and BMI were between the first and third tertiles. CART levels differed significantly across tertiles (all pairwise comparisons *p* < 0.001). When GD patients were evaluated, anti-thyroid peroxidase antibody (Anti-TPO) (*p* = 0.007) and TRAb (*p* = 0.015) levels showed significant differences among CART tertiles. No significant differences were observed among tertiles with respect to other clinical and laboratory parameters (Table 4).

**Figure 2:**
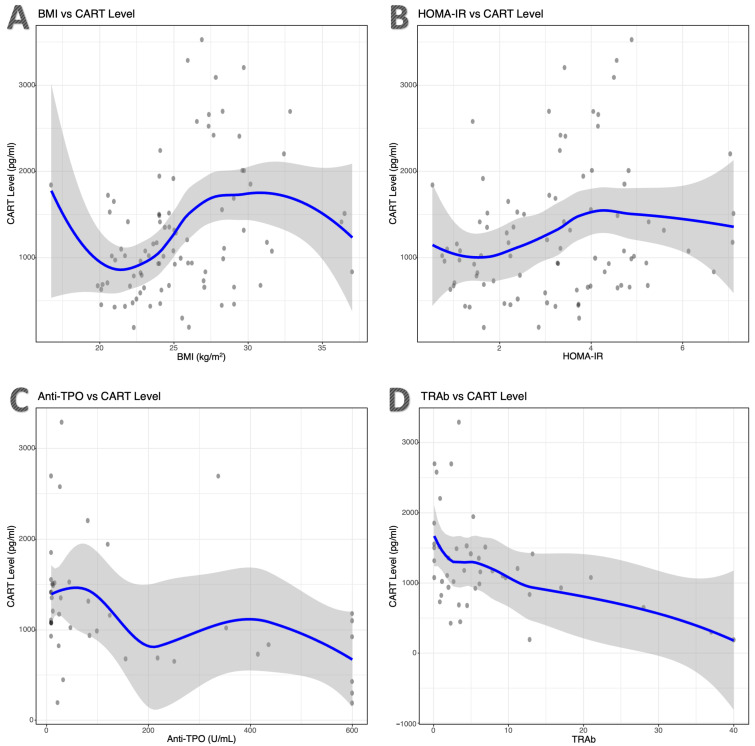
Correlation between serum cocaine- and amphetamine-regulated transcript (CART) levels and clinical parameters. Scatter plots illustrate the relationships between serum CART levels and (**A**) body mass index (BMI) and (**B**) homeostatic model assessment of insulin resistance (HOMA-IR) in the overall study population, as well as (**C**) anti-thyroid peroxidase antibody (Anti-TPO) and (**D**) thyrotropin receptor antibody (TRAb) in patients with Graves’ disease. Associations were assessed using Spearman’s rank correlation analysis. The blue line represents the LOESS (locally estimated scatterplot smoothing) curve, and the grey shaded area indicates the 95% confidence interval.

In correlation analyses, serum CART levels showed significant positive correlations with BMI (ρ = 0.369; *p* < 0.001) and HOMA-IR (ρ = 0.212; *p* = 0.048). In analyses performed within the GD group, significant negative correlations were observed between CART levels and Anti-TPO (ρ = −0.446; *p* = 0.002) and TRAb (ρ = −0.441; *p* = 0.003). No significant associations were found between serum CART levels and the other parameters (Figure 2).

## 3. Discussion

### 3.1. Basic Biology and Physiological Roles of CART

CART is a neuropeptide widely expressed in the central and peripheral nervous systems and is associated with energy balance, autonomic regulation, and neuroimmune communication. Derived from the CARTPT gene and processed into biologically active peptides by prohormone convertases, CART is mainly present in humans in the CART42–89 and CART49–89 forms [9]. Although it has been linked to anorexigenic signaling within hypothalamic circuits, its expression across different neuronal subpopulations and peripheral neural networks suggests that its effects may be context- and tissue-specific [10,11]. Experimental studies indicate that CART is involved not only in central mechanisms but also in enteric and autonomic neural networks and may participate in the modulation of peripheral immune responses via vagal pathways [12,13,14]. Taken together, existing experimental and review data indicate that CART has a broad spectrum of physiological effects that extend beyond central appetite control and intersect with metabolic and immune systems; this feature provides a biological basis for relating changes in CART levels to observations in systemic diseases [9,15].

### 3.2. Relationship Between CART and Obesity and BMI

Obesity and BMI are closely related to energy balance, and CART, an anorexigenic neuropeptide, has been implicated in the pathophysiology of obesity [10,16]. However, findings regarding the relationship between CART and obesity in the literature are heterogeneous. While coding region variants of the CART gene have been reported not to show a significant association with early-onset obesity [17], experimental and review studies indicate that CART expression may vary in relation to leptin signaling and metabolic status [11,13]. In the present study, the positive correlation observed between serum CART levels and BMI is consistent with previous reports suggesting that circulating levels of anorexigenic neuropeptides may increase in conditions of obesity and energy imbalance [15,16]. In this context, our findings suggest that CART may be part of adaptive processes related to metabolic burden rather than having a causal role in obesity.

### 3.3. Relationship Between CART and Autoimmunity and Inflammation

The interaction between the neuroendocrine system and the immune response has gained increasing importance in the pathogenesis of autoimmune diseases; in this context, CART is considered a potential modulator involved in neuroimmune communication beyond metabolic regulation. In experimental models of inflammation, CART expression has been reported to exhibit variable responses at both central and peripheral levels during systemic inflammatory stimuli [18]. Strong evidence for a direct interaction between CART and the immune system has been obtained from experimental studies demonstrating that CART-expressing vagal projections originating from the caudal dorsal motor nucleus directly innervate the spleen. These studies showed that CART release was associated with suppressive effects on inflammatory cytokine responses in splenocytes, that enhancement of CART signaling reduced inflammation, and that its blockade increased inflammatory responses [14]. Consistent with this experimental framework, the inverse associations observed in our study between serum CART levels and TRAb and Anti-TPO in the GD group, as well as the significant differences in autoantibody levels across CART tertiles, indicate that interindividual variability in CART levels may change in parallel with autoimmune burden. Although these findings do not establish a causal relationship, they suggest that CART may exhibit biomarker-like behavior associated with autoimmune activity in GD.

### 3.4. CART and the Thyroid Gland and the HPT Axis

In the experimental literature, the relationship between CART and the HPT axis is considered within a framework of context-dependent modulation that is sensitive to physiological conditions, rather than a fixed and unidirectional regulatory effect. At the hypothalamic level, CART signaling has been reported to vary in parallel with the feedback set point of the HPT axis under conditions such as fasting, energy balance, and environmental stress; depending on these conditions, its effects on TRH neurons may emerge in different directions [5,19,20,21].

Experimental evidence further supports an interaction between thyroid status and hypothalamic CART expression. In an animal study, hyperthyroidism induced a significant reduction in CART mRNA levels specifically in the PVN of the hypothalamus, whereas no change was observed in other nuclei such as the arcuate or dorsomedial nuclei [22]. Notably, CART-expressing neurons in the PVN have been shown to co-localize with TRH-producing neurons, implying that CART may participate in intrahypothalamic regulatory mechanisms of the HPT axis rather than directly reflecting peripheral thyroid hormone levels.

Consistent with this, anatomical and functional data indicate that although CART can be co-released with TRH, circulating CART levels do not directly reflect TSH secretion [5,21]. In the present study, the absence of a significant association between serum CART levels and TSH or thyroid hormones suggests that CART may be related to neuroendocrine adaptations developing in the setting of hyperthyroidism rather than to the hormonal outputs of the HPT axis. Therefore, at the clinical level, serum CART measurements should be considered not as a direct indicator of HPT axis activity but as a relational marker sensitive to physiological and pathophysiological context.

In addition to central mechanisms, anatomical studies have demonstrated the presence of CART-immunoreactive elements within thyroid tissue, particularly in parafollicular C-cells [23,24]. These findings suggest that CART may also exert local modulatory effects within the thyroid gland itself. However, whether circulating CART levels reflect such local thyroid expression remains unclear. Taken together, experimental and anatomical data indicate that the relationship between CART and thyroid status is complex and may involve both central and peripheral components.

### 3.5. Metabolic and Immune Heterogeneity in Graves’ Disease

Although GD is defined by a common autoimmune mechanism, it exhibits marked clinical, metabolic, and immunological heterogeneity; the severity of thyroid hormone excess, autoantibody profiles, and systemic effects may vary substantially among patients. At the immune level, while TRAb is the principal determinant of the disease, TPOAb and TgAb levels have been reported to be differently associated with tissue-level inflammation and clinical phenotypes, suggesting that the immune response in GD is not uniform [25,26]. From a metabolic perspective, the severity of the hypermetabolic state accompanying hyperthyroidism has been shown to be related not only to thyroid hormone levels but also to concomitant inflammatory activity [27,28]. In the present study, the absence of a direct association between serum CART levels and thyroid hormones or autoantibodies is consistent with this heterogeneous nature of GD. In this context, the finding that serum CART levels decreased with increasing ultrasound heterogeneity in GD patients classified according to thyroid ultrasonography suggests that CART may be related to tissue-level inflammatory and neuroendocrine heterogeneity rather than reflecting a single hormonal parameter in GD.

Recent spatial and molecular analyses of autoimmune thyroid disease have demonstrated marked intrathyroidal heterogeneity in immune cell infiltration and gene expression patterns, supporting the concept that ultrasound heterogeneity may reflect underlying tissue-level immune architecture rather than solely hormone excess [27]. In addition, correlations between ultrasound features and specific autoantibody profiles in GD have been previously reported, further indicating that imaging-based heterogeneity may represent biologically meaningful variation in the immunopathological activity of GD [25].

### 3.6. Serum CART Measurements and Clinical Studies

Although the physiological effects of CART have largely been defined through central and local neuroendocrine circuits, studies measuring serum CART levels in various clinical conditions have increased in recent years. Available clinical data indicate that circulating CART levels may vary across different disease groups; however, these variations should be interpreted in conjunction with metabolic, neuroendocrine, or stress-related contexts rather than as reflections of a single hormonal axis [9,29]. In studies examining psychiatric and behavioral conditions, serum CART levels have been reported to be lower compared with healthy individuals, but these differences have not shown consistent linear relationships with symptom severity or accompanying hormonal parameters [30,31]. Similarly, endocrine- or metabolism-focused studies have emphasized that serum CART measurements exhibit high interindividual variability and do not demonstrate direct parallelism with classical hormone levels [32]. These heterogeneous findings limit the interpretation of serum CART levels as standalone markers reflecting the activity of a specific physiological axis and instead suggest that they should be considered as components of the underlying neuroendocrine and metabolic state [9,29]. In this study, the evaluation of serum CART levels together with thyroid hormones and autoimmune markers provides an approach that is consistent with this context-sensitive and relational framework described in the existing clinical literature.

### 3.7. Limitations

This study has several limitations. First, the single-center design and relatively small sample size may limit the generalizability of the findings. However, considering the exploratory nature of the study, these results should be regarded as hypothesis-generating. Second, serum CART levels were measured at a single time point, which does not allow evaluation of longitudinal changes or treatment-related dynamics. Therefore, the relationship between temporal changes in CART levels and disease course or treatment response could not be determined within the scope of this study. Third, although the physiological effects of CART are known to occur largely through central and local neuroendocrine circuits, it remains unclear to what extent circulating CART levels reflect tissue-level expression or central activity. This represents an important factor limiting the biological interpretation of serum CART measurements. In addition, the semi-quantitative classification of thyroid ultrasound heterogeneity may be considered operator-dependent, as no standardized international scoring system exists for grading diffuse parenchymal heterogeneity in GD. Although ultrasonographic examinations were performed by a single experienced physician to minimize variability, interobserver reproducibility was not formally assessed. Finally, inflammatory cytokine profiles or other neuroimmune markers were not evaluated concurrently in this study; therefore, associations between serum CART levels and inflammatory activity were interpreted through indirect indicators. Future studies with larger sample sizes, multicenter designs, and longitudinal follow-up are expected to elucidate the clinical and biological role of CART in GD in greater detail.

## 4. Materials and Methods

### 4.1. Study Design and Participants

This single-center, prospective study was conducted between 1 October 2024, and 30 September 2025, in the Internal Medicine and Endocrinology Outpatient Clinic. A total of 44 patients aged 18–70 years with a diagnosis of GD were consecutively enrolled. In addition, 44 healthy individuals with similar age and sex characteristics were included as the control group. Individuals with autoimmune diseases other than thyroid disease, systemic inflammatory diseases, metabolic diseases that could affect inflammation, or signs of active infection at the time of evaluation were excluded from the study. None of the participants included in the study were using immunomodulatory or immunosuppressive medications that could affect the immune system.

At the time of blood sampling, all patients were receiving methimazole therapy. GD patients were classified according to their biochemical thyroid functional status at the time of sampling as euthyroid or hyperthyroid. Euthyroidism was defined as fT3 and fT4 levels within the laboratory reference range, whereas hyperthyroidism was defined as fT3 and/or fT4 levels above the upper reference limit. TSH was not used as a sole classification criterion, as it may show delayed normalization under antithyroid treatment. Disease duration was calculated from the time of initial diagnosis.

The study protocol was approved by the KTO Karatay University Faculty of Medicine Non-Drug and Non-Medical Device Research Ethics Committee (decision no: 2024/006, date: 26 September 2024). The study was conducted in accordance with the principles of the Declaration of Helsinki, and written informed consent was obtained from all participants.

### 4.2. Clinical and Laboratory Evaluation

For the diagnosis of GD, serum TSH, fT3, fT4, anti-thyroglobulin antibody (Anti-TG), Anti-TPO, and TRAb levels were measured in all patients and in the control group. Thyroid ultrasonography was performed by a specialist physician with more than 15 years of experience. All examinations were performed using the same ultrasound device under standardized operating conditions. Disease duration was recorded for the patients. Height, weight, and BMI measurements were obtained for all participants. To evaluate obesity, prediabetes, and type 2 diabetes mellitus, fasting plasma glucose, insulin levels, HbA1c, and HOMA-IR were assessed. Only individuals without any known disease were included in the control group; those with a BMI > 30 kg/m^2^, insulin resistance, impaired fasting glucose or a diagnosis of diabetes mellitus, as well as those with another autoimmune thyroid disease, were excluded from the study.

### 4.3. Thyroid Ultrasound Classification

According to thyroid ultrasonography findings, GD patients were classified based on diffuse parenchymal echogenicity. As no universally accepted and validated scoring system exists for grading diffuse parenchymal heterogeneity in GD, a semi-quantitative classification was applied, consistent with previously published approaches in autoimmune thyroid disease [33,34].

- Mild heterogeneity: Mild enlargement of the thyroid gland with echogenicity similar to normal thyroid tissue.
- Moderate heterogeneity: Multiple hypoechoic foci involving less than one-third of the thyroid parenchyma.
- High heterogeneity: Markedly hypoechoic thyroid tissue, with echogenicity similar to or lower than that of the anterior neck muscles.

Thyroid ultrasonography was also performed in all individuals in the control group, and normal findings were confirmed.

### 4.4. Measurement of Serum CART Level

Serum CART levels were measured using a commercial human CART kit based on the sandwich enzyme-linked immunosorbent assay (ELISA) method (Atlas Biotechnology, Ankara, Turkey; Catalog No: ABT2504Hu). The assay measurement range was 31.25–2000 pg/mL, and the analytical sensitivity was 18.75 pg/mL. According to the manufacturer’s data, the intra-assay and inter-assay coefficients of variation were below 10%. All samples were analyzed in duplicate, and absorbance values were measured at a wavelength of 450 nm using a microplate reader. CART concentrations were calculated from a standard curve generated for each assay. Serum samples with concentrations above the measurement range were reanalyzed after appropriate dilution in accordance with the manufacturer’s instructions.

### 4.5. Statistical Analysis

Statistical analyses were performed using Jamovi software (version 2.7.6.0; The jamovi project, Sydney, Australia) and IBM SPSS Statistics (version 27.0; IBM Corp., Armonk, NY, USA). The normality of continuous variables was assessed using the Shapiro–Wilk test in conjunction with histograms and Q–Q plots. Normally distributed data are presented as mean ± standard deviation, whereas non-normally distributed data are presented as median and interquartile range (25th–75th percentile). Categorical variables are expressed as counts and percentages. Comparisons between two independent groups were conducted using the independent samples *t*-test for normally distributed variables and the Mann–Whitney U test for non-normally distributed variables. For comparisons of three or more groups, one-way analysis of variance (ANOVA) was applied for normally distributed data, and the Kruskal–Wallis test was used for non-normally distributed data. When statistical significance was detected in overall analyses, pairwise comparisons were performed using the Tukey test following ANOVA and the Dwass–Steel–Critchlow–Fligner post hoc test following the Kruskal–Wallis test. As serum CART levels did not show a normal distribution, associations between CART and clinical, hormonal, autoimmune, and metabolic parameters were evaluated using Spearman correlation analysis. Correlation analyses were performed only in the GD group. Serum CART tertiles were defined based on the distribution of CART levels in the entire study population. In comparisons among CART tertiles, appropriate parametric or non-parametric tests were applied according to data distribution. Categorical variables were compared using the chi-square test or Fisher’s exact test. All statistical analyses were performed as two-sided, and a *p* value < 0.05 was considered statistically significant.

## 5. Conclusions

In this study, serum CART levels were evaluated in GD, and the associations of CART with thyroid hormones, autoimmune markers, and metabolic parameters were examined. Our findings indicate that serum CART levels are not directly associated with thyroid hormones or outputs of the HPT axis; however, they may be related to BMI and autoimmune burden. This suggests that, in GD, CART may be associated with heterogeneous metabolic and neuroendocrine adaptations rather than reflecting a single hormonal or immune axis. These findings indicate that the clinical interpretation of serum CART measurements should be approached within a context-sensitive and relational framework and that they have a hypothesis-generating character for future studies.

## Figures and Tables

**Table 1:** Comparison of the continuous and categorical variables between control and disease groups.

Parameters	Total *n* = 88	Control Group *n* = 44	Graves’ Disease *n* = 44	*p* Value
Gender, female	55 (62.5)	27 (61.4)	28 (63.6)	0.826
Hyperthyroidism	16 (18.2)	16 (36.4)	0 (0.0)	**<0.001**
Co-morbidities	0 (0.0)	0 (0.0)	0 (0.0)	—
Thyroid ultrasound findings				**<0.001**
Mild heterogeneity	17 (19.3)	0 (0.0)	17 (38.6)	
Moderate heterogeneity	18 (20.5)	0 (0.0)	18 (40.9)	
High heterogeneity	9 (10.2)	0 (0.0)	9 (20.5)	
Normal findings	44 (50)	44 (100.0)	0 (0.0)	
Age, years	38 ± 11	37 ± 10	40 ± 12	0.180
Height, cm	165 ± 8.32	167 ± 7.82	164 ± 8.65	0.115
Weight, kg	68.0 (63.0–75.3)	66.0 (63.0–70.0)	69.0 (63.0–77.0)	0.213
BMI, kg/m^2^	24.7 (22.8–27.9)	24.1 (22.0–27.2)	25.3 (23.8–28.3)	**0.044**
FBG, mg/dL	90.0 (81.8–98.0)	88.0 (81.8–94.0)	90.5 (81.8–107)	0.187
HbA1c, %	5.50 (5.10–5.90)	5.40 (5.00–5.90)	5.55 (5.27–5.90)	0.193
Insulin, mU/L	15.2 (8.38–18.6)	15.2 (8.90–18.0)	15.3 (7.75–19.1)	0.602
HOMA-IR	3.27 (1.74–4.32)	3.27 (2.15–4.00)	3.21 (1.71–4.67)	0.325
fT3, ng/L	3.11 (2.68–3.90)	2.90 (2.35–3.52)	3.52 (2.92–8.15)	**<0.001**
fT4, ng/L	12.1 (10.6–14.3)	11.4 (10.1–12.7)	13.9 (11.4–20.4)	**<0.001**
TSH, mU/L	1.31 (0.06–2.67)	2.09 (1.40–3.04)	0.06 (0.01–1.20)	**<0.001**
Anti-TG, U/mL	81.8 (17.7–300)	—	81.8 (17.7–300)	—
Anti-TPO, U/mL	31.3 (11.2–226)	—	31.3 (11.2–226)	—
TRAb, IU/L	3.83 (1.12–8.21)	—	3.83 (1.12–8.21)	—
CART level, pg/mL	1076 (686–1579)	965 (657–1859)	1133 (901–1504)	0.553

The bold values in the table indicate statistical significance. Anti-TG: anti-thyroglobulin, Anti-TPO: anti-thyroid peroxidase, BMI: body mass index, FBG: fasting blood glucose, HbA1c: hemoglobin A1c, HOMA-IR: homeostatic model assessment of insulin resistance score, TRAb: thyrotropin receptor antibody, TSH: thyroid-stimulating hormone, CART: cocaine- and amphetamine-regulated transcript.

**Table 2:** Clinical and laboratory characteristics according to thyroid ultrasound heterogeneity.

Parameters	Mild Heterogeneity *n* = 15	Moderate Heterogeneity *n* = 16	High Heterogeneity *n* = 13	*p* Value
Gender, female	10 (66.7)	11 (68.8)	7 (53.8)	0.677
Hyperthyroidism	4 (26.7)	9 (56.3)	3 (23.1)	0.114
Age, years	39.2 ± 13.8	38.4 ± 13.3	42.1 ± 9.3	0.714
Height, cm	164 ± 7.5	162 ± 9.7	166 ± 8.5	0.421
Weight, kg	73.8 ± 11.8	69.8 ± 9.8	68.5 ± 9.1	0.431
BMI, kg/m^2^	26.6 (24.5–29.0)	25.0 (23.8–28.8)	24.7 (22.3–26.0)	0.253
FBG, mg/dL	91.0 (87.0–101)	95.5 (82.8–109)	85.0 (79.0–107)	0.556
HbA1c, %	5.80 (5.50–6.30)	5.40 (5.15–5.70)	5.50 (5.20–5.80)	0.070
Insulin, mU/L	18.0 (8.09–22.6)	15.3 (10.7–18.2)	10.3 (7.47–16.7)	0.446
HOMA-IR	4.00 (1.73–5.07)	3.58 (2.12–4.62)	2.31 (1.66–3.74)	0.416
fT3, ng/L	3.51 (2.96–4.18)	6.26 (3.00–9.37)	3.45 (2.90–4.70)	0.685
fT4, ng/L	12.7 (11.4–14.9)	15.6 (10.1–25.9)	13.9 (12.8–15.8)	0.750
TSH, mU/L	0.27 (0.01–1.41)	0.02 (0.01–0.43)	0.51 (0.01–1.76)	0.352
Anti-TG, U/mL	121 (16.6–196)	46.1 (18.1–300)	110 (45–532)	0.467
Anti-TPO, U/mL	26.1 (10.6–81.6)	45.9 (12.4–177)	155 (10.4–353)	0.531
TRAb, IU/L	0.88 (0.13–4.54)	4.87 (1.97–8.21)	9.60 (3.41–21.00)	0.003 *
CART level, pg/mL	1414 (1127–1879)	1165 (1001–1515)	678 (426–1075)	<0.001 *

The bold values in the table indicate statistical significance. Anti-TG: anti-thyroglobulin, Anti-TPO: anti-thyroid peroxidase, BMI: body mass index, FBG: fasting blood glucose, HbA1c: hemoglobin A1c, HOMA-IR: homeostatic model assessment of insulin resistance score, TRAb: thyrotropin receptor antibody, TSH: thyroid-stimulating hormone, CART: cocaine- and amphetamine-regulated transcript. * Post hoc pairwise comparisons were performed using the Dwass–Steel–Critchlow–Fligner test. For CART levels, significant differences were observed between the high heterogeneity group and both the mild (*p* = 0.002) and moderate (*p* = 0.006) heterogeneity groups. For TRAb levels, significant differences were detected between the mild and moderate heterogeneity groups (*p* = 0.042) and between the mild and high heterogeneity groups (*p* = 0.007).

**Table 3:** Clinical and laboratory parameters according to disease duration.

Parameters	≤6 Months *n* = 16	6–24 Months *n* = 14	>24 Months *n* = 14	*p* Value
Gender, female	9 (56.3)	11 (78.6)	8 (57.1)	0.371
Hyperthyroidism	3 (18.8)	8 (57.1)	5 (35.7)	0.093
Thyroid ultrasound findings				0.255
Mild heterogeneity	3 (18.8)	4 (28.6)	8 (57.1)	
Moderate heterogeneity	7 (43.8)	6 (42.9)	3 (21.4)	
High heterogeneity	6 (37.5)	4 (28.6)	3 (21.4)	
Age, years	40.9 ± 13.6	37.7 ± 10.1	40.4 ± 13.2	0.785
Height, cm	163.8 ± 9.12	160.9 ± 7.85	167.2 ± 8.26	0.147
Weight, kg	68.0 (63.8–75.5)	74.5 (62.3–82.3)	70.0 (64.0–76.8)	0.820
BMI, kg/m^2^	24.8 (24.0–27.0)	27.2 (24.3–31.5)	24.5 (23.0–27.9)	0.220
FBG, mg/dL	86.5 (80.5–105)	107 (84.3–110)	90.0 (84.5–97.3)	0.349
HbA1c, %	5.55 (5.27–5.73)	5.85 (5.25–6.27)	5.50 (5.30–5.85)	0.484
Insulin, mU/L	13.3 (7.64–18.2)	17.9 (9.04–24.0)	14.1 (8.43–17.7)	0.477
HOMA-IR	2.70 (1.59–4.18)	4.53 (2.00–5.91)	2.70 (1.77–4.44)	0.387
fT3, ng/L	3.08 (2.79–4.29)	4.70 (2.96–10.3)	3.78 (3.25–6.43)	0.263
fT4, ng/L	12.4 (9.28–16.1)	14.9 (11.3–29.7)	14.4 (13.5–17.1)	0.206
TSH, mU/L	0.60 (0.01–2.05)	0.01 (0.01–0.94)	0.04 (0.01–0.34)	0.200
Anti-TG, U/mL	196 (35.7–469)	82.0 (16.9–322)	48.0 (16.8–159)	0.494
Anti-TPO, U/mL	25.5 (10.1–174)	104 (17.5–559)	25.9 (12.6–94.6)	0.304
TRAb, IU/L	2.88 (1.79–7.17)	5.92 (4.20–10.8)	1.44 (0.28–5.66)	0.044 *
CART level, pg/mL	1450 (1064–2102)	1006 (857–1173)	1092 (754–1464)	0.054

The bold values in the table indicate statistical significance. Anti-TG: anti-thyroglobulin, Anti-TPO: anti-thyroid peroxidase, BMI: body mass index, FBG: fasting blood glucose, HbA1c: hemoglobin A1c, HOMA-IR: homeostatic model assessment of insulin resistance score, TRAb: thyrotropin receptor antibody, TSH: thyroid-stimulating hormone, CART: cocaine- and amphetamine-regulated transcript. * Post hoc pairwise comparisons were performed using the Dwass–Steel–Critchlow–Fligner test. A significant difference in TRAb levels was observed between the 6–24-month group and the >24-month group (*p* = 0.047). No significant differences were observed between the ≤6-month group and the other groups.

**Table 4:** Clinical and biochemical characteristics according to CART tertiles.

Parameters	Tertile 1 *n* = 29	Tertile 2 *n* = 29	Tertile 3 *n* = 30	*p* Value
Gender, female	21 (72.4)	19 (65.5)	15 (50.0)	0.189
Graves’ Disease	11 (37.9)	19 (65.5)	14 (46.7)	0.099
Hyperthyroidism	4 (13.8)	7 (24.1)	5 (16.7)	0.573
Age, years	39.1 ± 9.58	38.8 ± 10.36	36.7 ± 12.99	0.669
Height, cm	169.1 ± 8.20	165.2 ± 7.37	161.8 ± 7.96	0.003 *
Weight, kg	68.0 (66.0–76.0)	66.0 (63.0–72.0)	66.0 (63.0–77.0)	0.494
BMI, kg/m^2^	27.3 (24.0–29.4)	24.7 (23.6–26.0)	22.9 (22.1–26.8)	0.010 *
FBG, mg/dL	90.0 (84.0–98.0)	88.0 (82.0–99.0)	89.0 (78.3–94.8)	0.382
HbA1c, %	5.70 (5.30–6.00)	5.40 (5.20–6.00)	5.40 (5.00–5.68)	0.203
Insulin, mU/L	16.4 (13.7–18.3)	13.4 (7.65–19.2)	13.6 (7.81–18.1)	0.171
HOMA-IR	3.44 (3.08–4.50)	3.04 (1.57–4.72)	2.93 (1.66–3.88)	0.097
fT3, ng/L	3.08 (2.80–3.80)	3.17 (2.67–4.10)	3.11 (2.54–3.92)	0.889
fT4, ng/L	12.1 (10.2–14.0)	11.4 (10.3–14.6)	12.9 (11.6–14.4)	0.343
TSH, mU/L	1.90 (0.55–3.00)	0.72 (0.01–1.79)	1.52 (0.54–2.19)	0.231
CART level, pg/mL	2009 (1651–2578)	1076 (986–1206)	641 (455–686)	<0.001 *
**Parameters**	**Tertile 1** ***n*** ** = 11**	**Tertile 2** ***n*** ** = 19**	**Tertile 3** ***n*** ** = 14**	***p*** ** Value**
Anti-TG, U/mL	73.0 (20.7–192)	36.2 (15.8–423)	110 (49.5–483)	0.503
Anti-TPO, U/mL	15.7 (11.5–44.5)	26.4 (9.05–105)	251 (93.8–518)	0.007 *
TRAb, IU/L	0.88 (0.14–3.40)	5.83 (2.01–9.32)	4.49 (2.85–20.4)	0.015 *

The bold values in the table indicate statistical significance. Anti-TG: anti-thyroglobulin, Anti-TPO: anti-thyroid peroxidase, BMI: body mass index, FBG: fasting blood glucose, HbA1c: hemoglobin A1c, HOMA-IR: homeostatic model assessment of insulin resistance score, TRAb: thyrotropin receptor antibody, TSH: thyroid-stimulating hormone, CART: cocaine- and amphetamine-regulated transcript. * Post hoc pairwise comparisons were performed using the Dwass–Steel–Critchlow–Fligner test. For height, a significant difference was observed between tertile 1 and tertile 3 (*p* = 0.002). For BMI, a significant difference was detected between tertile 1 and tertile 3 (*p* = 0.019). Anti-TPO levels differed significantly between tertile 1 and tertile 3 (*p* = 0.004), whereas no significant differences were observed between the other pairwise comparisons. TRAb levels showed significant differences between tertile 1 and tertile 3 (*p* = 0.042) and between tertile 2 and tertile 3 (*p* = 0.032). CART levels demonstrated significant differences across all pairwise tertile comparisons (all *p* < 0.001).

## Data Availability

The data presented in this study are not publicly available due to ethical and privacy restrictions related to participant confidentiality but are available from the corresponding author upon reasonable request.

## Abbreviations

The following abbreviations are used in this manuscript:

BMI	Body mass index
CART	Cocaine- and amphetamine-regulated transcript
ELISA	Enzyme-linked immunosorbent assay
FBG	Fasting blood glucose
fT3	Free triiodothyronine
fT4	Free thyroxine
GD	Graves’ disease
HbA1c	Glycated hemoglobin
HOMA-IR	Homeostatic Model Assessment of Insulin Resistance
HPT axis	Hypothalamic–pituitary–thyroid axis
PVN	Paraventricular nucleus
TRAb	Thyrotropin receptor antibody
TRH	Thyrotropin-releasing hormone
TSH	Thyroid-stimulating hormone
Anti-TG	Anti-thyroglobulin antibody
Anti-TPO	Anti-thyroid peroxidase antibody
